# Supplementary material for: Are carbon emissions trading and green financial instruments synergistic? -Comprehensive quantitative research based on content analysis
Source: PLoS One. 2024 Mar 7;19(3):e0298601. doi: 10.1371/journal.pone.0298601 (PMC10919601; doi:10.1371/journal.pone.0298601)
Supplement: S1 Data — (ZIP) [file pone.0298601.s001.zip › Data/地级市Z_F工作报告.zip!/╕╩╦α/╠∞╦«╩╨/└┤╫╘╣1⁄2╓┌║┼í╛┬φ┐╦╩2╛▌═°í┐_2016.docx]

各位代表:

现在,我代表市人民政府向大会报告工作,请与《天水市国民经济和社会发展第十三个五年规划纲要(草案)》一并审议,并请各位政协委员和列席人员提出意见建议。

(一)“十二五”时期的重点工作和2015年的工作回顾

“十二五”时期,是我市发展进程中很不平凡的五年。五年来,面对错综复杂的经济环境和艰巨繁重的改革发展稳定任务,在省委、省政府和市委的正确领导下,市政府主动适应新常态,积极应对新变化,牢牢把握“主动作为、稳中求进、赶超进位、务求实效”的工作总基调,团结带领全市人民,抢抓机遇,攻坚克难,统筹推进稳增长、促改革、调结构、惠民生、防风险各项工作,较好地完成了“十二五”规划确定的目标任务,全市经济社会呈现持续稳定发展的良好态势。

五年来,我们更加注重稳增长促发展,经济发展成效明显,综合实力显著增强。“十二五”时期,全市大部分经济指标增速高于全省平均水平,主要经济指标增幅在全省排名持续前移。2015年全市生产总值达到553.8亿元,在2010年基础上跨越两个百亿元台阶,年均增长10.9%,经济总量在全省的位次从2013年的第5位上升到去年的第3位,仅次于兰州市、庆阳市。累计完成固定资产投资2227亿元,投资规模是“十一五”时期的3倍,年均增长23.2%。社会消费品零售总额达到262.42亿元,是2010年的近2倍,年均增长14.3%。财政收入和财政支出实现“双翻番”,大口径财政收入突破百亿元,达到110.22亿元,是2010年的2.34倍,年均增长18.6%;财政支出达到246.5亿元,是2010年的2.04倍,年均增长15.3%。城乡居民收入连续五年高于生产总值增幅,农村居民人均可支配收入达到6006元,比2010年翻了一番,年均增长16.3%;城镇居民人均可支配收入达到20809元,是2010年的1.8倍,年均增长12.6%。金融机构存款余额突破1000亿元,达到1033.4亿元,是2010年的2.2倍;贷款余额642.6亿元,是2010年的近3倍;直接融资58.6亿元,是“十一五”时期的12.6倍。粮食总产连续八年超过100万吨,实现“十二连丰”。

五年来,我们更加注重转方式调结构,特色产业发展壮大,转型升级步伐加快。三次产业结构由“十一五”末的20:38:42调整为17.6:33.5:48.9,产业结构更趋优化。全市“48113”现代农业计划稳步推进,果品、蔬菜、畜牧业总产值分别达到80亿元、58亿元、65亿元,是“十一五”末的3.3倍、2.2倍和1.5倍;劳务收入迈上百亿元台阶,达到111.5亿元,较2010年增加48.5亿元,农业四大主导产业收入占农民收入比重超过80%。“工业强市”战略深入实施,规模以上工业增加值达到111亿元,比2010年翻了一番,年均增长13.7%;华天科技、长城电工、星火机床三大产业园区相继建成,天水卷烟厂易地技改、太极集团阿胶、娃哈哈、中材水泥、西电高压电工触头等项目建成投产,华天电子等8户企业列为全省战略性新兴产业骨干企业,创建国家级示范生产力促进中心2个,单位工业增加值能耗完成目标任务。我市被命名为“国家商标战略实施示范城市”,获批创建“全国质量强市示范城市”,有效商标总量达到2204件,中国驰名商标达到15件,新增甘肃名牌产品56个、总数达到77个,名牌产品新增数和保有量均居全省前列。第三产业提速发展,麦积山石窟列入世界文化遗产名录,伏羲庙等7个景区晋升为国家4A级景区,文化产业增加值年均增长30%,旅游综合收入年均增长29.7%。非公有制经济健康发展,占生产总值比重由“十一五”末的34.5%提高到49.6%。

五年来,我们更加注重抓项目扩投资,发展基础不断夯实,城乡面貌明显改观。以“3341”项目工程为抓手,累计实施亿元以上重大项目507个,天平铁路、十天高速、宝天高速过境段及街亭温泉出口、庄天二级公路、武山洛门至礼县二级公路、凯迪生物质能源发电、中卫至贵阳天然气管道天水段、750千伏输变电、社棠330千伏输变电等一批重大交通能源项目全面建成,宝兰客专天水段、国道310秦州至武山段升级改造、甘谷至麦积二级公路、城区引洮供水等项目加快建设。全市公路通车总里程突破1万公里,市县之间实现二级以上公路连接,4个县区通高速公路,所有乡镇和94.5%的建制村通沥青(水泥)路。加强城市基础设施建设,累计实施城建项目185项,总投资282.8亿元,建成96个,完成投资83亿元。建成城市主次干道26条、桥梁22座,新建改建广场4个,硬化亮化小巷道280条,新建改建公厕104座,新增绿地210万平方米,城市建成区面积达到56平方公里,常住人口城镇化率达到43.2%,较2010年提高11.3个百分点。

五年来,我们更加注重促改革增活力,体制机制更趋完善,对外开放继续扩大。政府机构改革全面完成,事业单位改革完成分类工作。取消、调整和下放行政审批事项188项,市级政府部门行政审批事项由2002年的1059项削减为163项,削减率84.6%。工商、质监、食药监、卫生系统、文化、广电等管理体制调整理顺。落实“营改增”试点政策,涉及的服务业领域减税面达97%。国资国企改革持续深化,农村综合改革稳步推进,流转土地95.1万亩,农村土地承包经营权确权登记颁证和甘谷县全国农村供销合作社综合改革试点进展顺利。“三证合一、一照一码”全面实行,市场主体达到10.7万户,是2010年的2.1倍,年均增长20.8%。天水海关办事处挂牌运营,2015年实现外贸进出口总额23.85亿元,是2010年的1.6倍,年均增长11%。交通银行、甘肃银行、浙商银行、长安银行、浦发银行、招商银行等12家金融机构入驻天水或设立办事机构,金融服务体系更加完善。对外交流全面开展,《关中—天水经济区发展规划》深入实施。开通天水至重庆、天津、大连等地航班,天水至西安形成每日三班“航空快线”,2015年旅客吞吐量突破6万人次,是2010年的11倍。公祭伏羲大典实现海峡两岸共祭,环青海湖自行车赛、中国龙舟公开赛、全国山地自行车锦标赛、麦积山自行车赛、国际传统射箭邀请赛等重大赛事成功举办,阿拉伯国家驻华使节天水行活动成果丰硕。加大招商引资力度,累计签约招商项目1087项,到位资金1179.63亿元,年均增长48.5%。

五年来,我们更加注重保基本兜底线,民生事业持续发展,社会大局和谐稳定。坚持每年办理一批民生实事,市级财政支出85%以上用于改善民生。扶贫攻坚取得重大突破,“十二五”期间减贫85.37万人,贫困发生率由43.3%下降到15.6%。实施农村饮水安全工程86处,解决了167.2万人饮水安全问题。建成农家店1035个,新建扩建商品配送中心10个,新建改造县乡农贸市场36个。实施科技项目872项,取得创新成果706项。学前教育三年毛入园率、九年义务教育巩固率、高中阶段毛入学率分别达到66.8%、87.5%、88.3%,较2010年分别提高36.7、16.3、15.6个百分点,实现乡镇中心幼儿园和2000人以上行政村幼儿园全覆盖,实施教育改薄项目2111个,完成投资12.23亿元,我市通过“两基”国检验收。完成乡镇社区体育惠民工程41个,村级农民健身工程1031个。市妇幼保健院迁建、秦州区医院迁建、天水全科医师临床培训基地和儿童病区综合楼、407医院综合楼基本完工,新农合参合率达到97.3%。建成乡镇综合文化站113个、村文化室375个、乡村舞台1784个、农家书屋(社区书屋)2557个、城市数字影院7个。《天水通史》、《魅力天水》出版发行,创排了《一画开天》、《麦积圣歌》、《大秦文公》、《轩辕大帝》、《陇上铁汉》、《关山月》、《邓宝珊将军》、《姜维》等精品剧目。城镇就业总量持续增加,累计新增就业33.62万人。社会保障体系日益健全,退休职工养老金平均待遇、城镇居民医保报销比例逐年提高,城市低保、农村低保、五保户供养标准分别比2010年提高85.6%、186.4%和120.5%。累计建设保障性住房7.22万套664.9万平方米,实施农村危房改造8.66万户,发放廉租住房补贴9.8万户16.3亿元。特大暴洪和群发性地质灾害恢复重建基本完成。成功创建全国双拥模范城“五连冠”。环境质量持续好转,安全生产形势稳定,民族宗教事务和顺,“平安天水”建设深入推进,社会大局和谐稳定。统计调查、档案史志、防震减灾、社会团体、妇女儿童、老龄老干、老年教育、残疾人事业和关心下一代等工作都取得了新的成绩。

五年来,我们更加注重转职能改作风,自身建设全面加强,政风行风持续好转。全面落实省委、省政府和市委各项决策部署,自觉依法接受市人大及其常委会的法律监督和工作监督、市政协及参加单位的民主监督、各界各方的群众监督和社会监督,全面执行人大决议,充分吸纳政协意见,五年办结人大代表意见建议854件、政协提案1221件。严格执行中央“八项”规定、国务院“约法三章”、省委“双十条”规定,认真落实政府系统党风廉政建设主体责任,巩固党的群众路线教育实践活动成果,扎实开展“三严三实”专题教育,持续推进“效能风暴”行动,有效整改“四风”和“不严不实”问题,政府自身建设持续加强。坚持依法行政,“六五”普法全面完成,全民法治意识明显增强。深入推进政务公开和电子政务,“三张清单一张网”建立并上线发布,行政审批事项网上“一站式”办结和联审联批积极推进,市公共资源交易中心逐步完善运行,政府及政府部门服务意识、服务水平不断提升。

各位代表,刚刚过去的2015年,我们迎难而上、主动作为,在适应新常态中认真落实市六届人大四次会议确定的工作任务,实现了“十二五”发展的圆满收官。

一是赶超进位成效明显。主要经济指标增幅在全省的位次进一步前移,生产总值同比增长9.2%,增速由2014年全省第7位前移到第2位;第一产业增长6.1%,由第3位前移到第1位;第二产业增长10%,由第4位前移到第2位;第三产业增长9.6%,由第8位前移到第7位;规模以上工业增加值增长10%,由第4位前移到第2位;固定资产投资增长12.1%,由第7位前移到第6位;社会消费品零售总额增长9.2%,由第2位前移到第1位;公共财政收入增长15.3%,由第9位前移到第3位;公共财政支出增长24.9%,由第8位前移到第1位;城镇居民人均可支配收入增长10%,与2014年位次持平,位居全省第6位;农村居民人均可支配收入增长12.5%,与2014年位次持平,位居全省第2位。全市主要经济指标增长幅度均高于全省平均水平,政府目标考核各项指标及部门行业性考核结果绝大部分位列全省市州前列。

二是精准扶贫扎实推进。制定出台“1+18”精准脱贫方案,在1034个贫困村开展“万名干部轮流驻村”活动,双联行动与扶贫攻坚融合推进。建立多渠道多元化投入机制,贫困村扶贫互助资金协会实现全覆盖,发放精准扶贫贷款44亿元,争取财政专项扶贫资金5.6亿元,整合项目资金48亿元,投入各类精准扶贫资金114亿元。建成贫困村通畅工程244个、794.3公里,标准化村卫生室634个,提前实现所有贫困村通沥青(水泥)路、标准化村卫生室全覆盖的目标。全年脱贫20万人,贫困人口减少到47.78万人。

三是重大项目全面推进。100个市列重大项目完成投资236.6亿元。天平铁路、十天高速等一些重大项目和张家川县城至恭门火车站二级公路、麦积党川至两当公路建成通车,藉口水厂主体完工,张家川49.5兆瓦风电项目并网发电,国道310秦州至武山段升级改造、甘谷至麦积二级公路、城区引洮供水、甘谷县陇东南汽配城、张家川富川水库、秦安小湾河水库等项目加快建设,天水机场迁建、曲溪城乡供水、平凉至天水高速公路等项目前期工作顺利推进,财政易地搬迁项目启动实施。招商引资成效明显,新签约项目243项,到位资金420.7亿元,同比增长34.5%。

四是经济建设稳步发展。工业经济逆势平稳运行,规模以上工业增加值同比增长10%。实施千万元以上工业项目165项,完成投资129.5亿元,天水烟用包装印刷生产线、大型客机改货机等项目基础工作有效推进。开发区扩区增容步伐加快,全国电工电器产业知名品牌示范区加快创建,国家智能电网输变电设备质量监督检验中心建成,铁路电缆、长控公司等企业出城入园有序推进,众兴菌业、成纪药业成功上市,风动机械在“新三板”上市,95户企业在区域性股权市场挂牌。企业自主创新能力增强,50件新产品新技术通过省级鉴定,我市被评为“2015年度全国十大质量魅力城市”,成为甘肃首次获此殊荣的城市。农村经济稳步发展,粮食总产达到126.95万吨,10个现代农业示范园区和100个科学发展示范点完成投资30.3亿元,新建果品基地19万亩,新增蔬菜面积4.8万亩,新发展规模养殖场100个,实现劳务收入111.5亿元。第三产业快速发展,武山洛门蔬菜批发市场迁建基本完成,天水商贸城、甘谷县浙江商贸城二期、秦安中国西部小商品城、天水城市蔬菜仓储分拣中心等项目加快实施,居然之家直营店落户天水,众创空间暨电子商务中心即将建成。天水文化旅游投资发展公司组建成立,麦积山温泉度假酒店、麦积区和秦州区两个滑雪场等项目建成运营,麦积山石窟艺术研究所项目、孙集文化小镇、峡门风情小镇等文化旅游项目加快建设,天水至兰州“麦积山号”旅游专列和天水至陇西“城乡文化列车”开通。全年接待游客2216.5万人次,旅游综合收入125.7亿元,分别增长33%和32.3%,我市被评为“中国最具魅力文化旅游城市”。

五是城市建设亮点纷呈。启动第四版城市总体规划修编。实施城建重大项目52项,总投资162亿元,完成投资36亿元。藉河南路西延段、成纪大道、马跑泉大道东延段、藉河孙家坪大桥、藉河山水一号大桥、渭河峡口大桥、渭河社棠大桥、渭河武山西关大桥、天水建筑科技中心等项目相继建成,藉河双桥大桥、藉河罗峪河口大桥、麦积区区府路西延段、甘肃建投天水总部经济城等项目加快建设,秦州大道、麦积山大道、成纪新城路网一期、藉河罗家沟大桥、天水固体垃圾处理循环产业园、麦积污水管网改造、高铁南站综合交通枢纽及站前广场、市体育中心、秦州热源厂、成纪新城地下净水厂、有轨电车试验段等项目启动实施,城区供热管网改造、秦州至三阳川隧道、藉河二十里铺大桥等项目前期工作有序推进。成纪新城输配水管网建成运行,市民吃水难问题得到缓解。五县县城建设步伐加快,甘谷大像山文化产业园、清水轩辕文化产业园等项目全面推进。房地产市场企稳回升,投资、建设、销售同比都有增长。城建民生工程顺利实施,城区绿化美化亮化步伐加快,马跑泉公园提升改造、藉河风情线一期延伸、西十里藉河生态公园进展顺利,全国“智慧城市”试点全面启动,我市荣获“中国十大最美城镇”称号。

六是人民生活持续改善。省列10项27件、市列10件民生实事全部办结。新增城镇就业10.3万人,城镇登记失业率为3.1%,居民消费价格指数涨幅为2%。城镇居民大病医疗保险启动实施,被征地农民养老保险取得重大突破,“五险合一”社会保险信息系统上线运行。开工建设保障性住房1.4万套104.34万平方米,主体竣工7341套,完成农村危旧房改造1.99万户。发放廉租住房补贴1.37万户2678.6万元、社会救助资金11.9亿元。争取国列、省列科技项目63项,取得科技成果86项,其中62项达到国内先进以上水平。“全面改薄”成效明显,县域义务教育均衡发展,市一中麦积校区、市一幼教学楼等项目建设进展顺利,市三中迁建项目启动实施。建成省级救灾仓库、儿童福利院、流浪未成年人救助保护中心等项目。建设乡村舞台821个、县级城市数字影院6座,完成农村电影放映3万多场次。人口自增率6.93‰。《幸福快车》、《苦乐村官》等影视剧拍摄完成。体育赛事接连不断,我市上榜“全国最具体育活力城市”。环保专项整治深入开展,环境空气质量不断改善。社会事业持续发展,社会管理不断加强,其他各项社会事业和民生事业有序推进。

各位代表,“十二五”时期,是我市经济总量增长最快、城乡面貌变化最大、人民群众得到实惠最多的五年。这些成绩的取得,是省委、省政府正确领导、亲切关怀的结果,是市委统揽全局、科学决策的结果,是市人大及其常委会、市政协及社会各界有效监督、倾力支持的结果,是全市广大干部群众及各族各界人士和衷共济、奋力拼搏的结果。在此,我代表市人民政府,向奋战在全市各条战线上的广大干部群众,向各民主党派、人民团体、工商联和无党派人士,向离退休老同志,向天水籍和天水工作过的在外人士,向驻市人民解放军、武警消防官兵和驻市单位,表示崇高的敬意和诚挚的感谢!

各位代表,在肯定成绩的同时,我们清醒地认识到,发展中还存在不少困难和问题:经济总量不大,战略性新兴产业比重低,服务业发展层次不高,保持经济稳定增长的难度较大;产业结构不尽合理,创新创业活力不足,发展质量和效益不高,调结构转方式的双重压力较大;城乡基础设施建设相对滞后,城镇建设与群众的期待还有相当大的差距,生态环境保护和节能减排降耗任务艰巨;农村贫困面大,贫困程度深,脱贫攻坚和产业培育的任务依然繁重;社会保障体系有待完善,基本公共服务供给还需提升,改善民生需要付出更大努力;发展环境不够宽松,办事难、效能低的问题在一些单位比较突出,作风建设永远在路上,等等。对这些问题,我们将采取有效措施,在工作中认真加以解决。

(二)“十三五”时期的发展目标和工作重点

“十三五”时期,是我市全面建成小康社会的决胜阶段,也是经济社会转型升级发展的关键时期和重要机遇期。我市经济社会发展中虽然存在不少困难与挑战,但更面临诸多重大机遇和利好政策。国家深入实施西部大开发、推进“一带一路”战略、建设关中—天水经济区以及全省三大战略平台等带来的政策和市场机遇多重叠加,并且随着“十三五”规划的出台实施,其效应将加速释放;中央城市工作会议提出在中西部地区发展一批城市群和区域中心城市,天水是甘肃能够列入国家层面城市群规划的两个城市之一,这对我们加快新型城镇化进程、建设区域中心城市是很好的机遇;国家深入推进供给侧结构性改革,重点去产能、去库存、去杠杆、降成本、补短板,有利于我们转换增长动力、推动产业升级、提高经济发展质量和效益。只要我们扬长避短、顺势而为,精心谋划、狠抓落实,就一定能够把各项事业发展推向新的历史阶段。

按照《中共天水市委关于制定国民经济和社会发展第十三个五年规划的建议》,“十三五”时期我市经济社会发展的指导思想是:全面贯彻落实中央各项方针政策、决策部署和习近平总书记系列重要讲话精神,按照省委、省政府和市委的部署要求,牢固树立创新、协调、绿色、开放、共享的发展理念,积极适应、把握和引领经济发展新常态,继续坚持“主动作为、稳中求进、赶超进位、务求实效”的工作原则,着力保持合理增长,着力调整优化结构,着力提高发展质量,着力深化改革开放,着力推进脱贫攻坚,着力保障改善民生,确保到2020年与全国全省一道全面建成小康社会,努力建设丝绸之路经济带历史文化名城、区域中心城市和现代化大城市。

经济社会发展的预期目标是:“十三五”期间,力争“实现四个高于、做到四个控制、跨上三个台阶”,即生产总值、固定资产投资、财政收入和城乡居民收入增幅高于全省平均水平;居民消费价格指数、人口自然增长率、城镇登记失业率和单位生产总值能耗控制在省上下达的指标以内;生产总值跨上800亿元台阶,城镇居民人均可支配收入跨上30000元台阶,农村居民人均可支配收入跨上10000元台阶。具体来说,生产总值年均增长8%,固定资产投资年均增长10%,社会消费品零售总额年均增长10%,公共财政预算收入年均增长8%,城镇居民人均可支配收入年均增长9%,农村居民人均可支配收入年均增长12%。

围绕以上目标,我们将重点抓好以下工作:

一、坚持精准聚焦施策,强化“一号工程”意识,坚决打赢精准脱贫攻坚决胜战

改善基本条件强基础。全面落实“853”挂图作业任务,深入实施“1+18”精准脱贫方案,建成农村公路通畅工程109条775公里,实现村村通沥青(水泥)路;解决99个贫困村、8.89万贫困人口饮水安全问题,实现村村通安全饮水;解决21个贫困自然村通动力电问题,实现村村通动力电;完成45个乡镇、2017个行政村广播电视有线网络建设,实现村村通有线广播电视;建设通讯基站4800座,实现贫困村4G互联网络全覆盖;实施危房改造5.4万户,完成所有贫困户危房改造任务;搬迁贫困群众2.11万户9万人,实现具有搬迁条件和意愿的贫困户应搬尽搬。

发展富民产业促增收。加大扶贫资金投入力度,市级按当年一般公共预算收入增量的10%以上,县区按20%以上增列专项扶贫预算。继续实施产业“精准扶贫专项贷款”工程,为贫困户提供5万元以内3年以下银行免抵押免担保贴息信贷资金。大力扶持贫困农户发展果品、蔬菜、畜牧、劳务等主导产业和中药材、马铃薯、花卉等特色优势产业,重视农村实用技术培训。“十三五”末,实现贫困户有需求劳动力技能培训全覆盖,每个贫困家庭至少有1人掌握1门致富技能,贫困村农民人均可支配收入达到10000元以上。

强化公共服务补短板。推动学前教育资源向贫困村倾斜,实现有实际需求的贫困村学前教育全覆盖。实施文化惠民扶贫项目,实现贫困村乡村舞台全覆盖。重视加强养老服务机构建设,实现贫困村养老机构、日间照料中心全覆盖。加强贫困乡村医生队伍建设,每个乡镇卫生院平均配备5名以上全科医生,贫困村村医全部具备乡村医生执业资格以上标准,提高符合条件的贫困村村医定额补助标准。“十三五”末,确保现行标准下农村贫困人口实现脱贫,贫困县区全部摘帽。

二、坚持创新驱动战略,加快产业转型升级,努力提升经济发展质量和效益

改造提升传统优势产业。深入实施“工业强市”战略,加快推进提质扩量工程,着力打造5户年销售收入上50亿元的产业化企业、10户年销售收入上10亿元的骨干性企业、100户以上年销售收入上亿元的成长性企业,争取10户以上企业通过直接上市或并购重组上市融资,推动机械制造、电工电器、电子信息、医药食品、建筑建材、能源化工等传统产业优化升级,打造年销售收入上百亿元的六大产业集群,逐步建设产值上千亿元的国家级开发区,力争“十三五”期间全市规模以上工业增加值年均增长9%。

扶持发展战略性新兴产业。加大华天电子等省市级战略性新兴产业骨干企业扶持力度,重点发展以高端数控机床、新一代集成电路、智能化输配电装备等为重点的先进装备制造产业企业,以“互联网+”、“大数据”、“云计算”等为重点的新一代信息产业企业,以现代中药、新型制剂等为重点的生物医药产业企业,积极发展新能源和清洁能源产业企业、新材料产业企业,培育经济发展新的增长点。实施创新驱动发展战略,培育10户以上国家和省级技术创新示范企业,建设大型电气传动系统与装备技术国家重点实验室、国家电线电缆质量监督检验中心,重视加强品牌建设,建成“全国质量强市示范城市”。大力发展非公经济,积极扶持民营企业和中小微企业发展,支持新型创业服务平台建设,实现众创空间县区全覆盖,促进大众创业、万众创新。

加快现代农业发展步伐。深入实施“48113”现代农业推进计划,稳定发展粮食生产,做大做强果品、蔬菜、畜牧、劳务“四大主导产业”,完善提升“十个现代农业示范园区”,全力办好“100个科学发展示范点”,持续推进“十个百万工程”,培育壮大“百强龙头企业”,着力打造“三条城乡一体化示范长廊”,提升现代农业发展水平。“十三五”末,农业增加值达到120亿元,年均增长5.5%;果品、蔬菜、畜牧、劳务四大主导产业均达到“百亿元产业”,力争总产值超过600亿元;培育1—2户上市龙头企业,农业产业化龙头企业销售收入达到100亿元。

提升现代服务业发展水平。加快发展电子商务,尽快建成天水众创空间暨电子商务中心,加强与阿里巴巴、京东、苏宁云商、纳世利等电商平台协作,发展多层次多门类和跨境电子商务,创建“国家级电子商务示范城市”,全市电子商务交易额年均增长30%以上,网络零售额年均增长35%以上。积极发展科技、金融、商务等生产性服务业和健康、养老、旅游等生活性服务业,“十三五”末,全市服务业增加值达到410亿元,年均增长9%。

促进文化旅游深度融合。深入实施华夏文明传承创新区“136”工程,加强陇东南“祖脉”文化旅游圈建设,着力打造羲皇故里·世界华人寻根祭祖圣地、人文天水·丝绸之路历史文化名城、陇上江南·中国休闲旅游生态家园三大品牌,办好关中—天水经济区、陇东南、陕甘川毗邻地区三大文化旅游联盟,推进中华伏羲文化城、大地湾国家考古遗址公园、麦积山文化旅游大景区项目建设,重点实施西部华昌城、养老基地及翠园文化产业园、士林不夜城、麦积百花小镇、秦风民俗园、秦州印象文景园、秦州南山片区文化旅游融合示范区、数字麦积山及游客中心、工业博物馆等项目,推动文化旅游融合发展。“十三五”末,全市文化产业增加值达到40亿元,旅游综合收入达到338亿元。

三、坚持项目带动战略,持续增加有效投资,不断夯实经济社会发展基础

构建区域交通枢纽。实施“6873”交通突破行动,公路方面,开工建设平凉至天水高速公路项目,加快推进甘谷至麦积二级公路、国道310秦州至武山段升级改造等项目,争取尽早启动静宁至天水高速、天水南绕城高速和陕西陇县至甘谷至通渭榜罗、秦安莲花经张家川至陕西陇县石槽沟公路等项目,加快实施农村公路通畅工程和重点景区旅游公路建设,“十三五”末力争实现“县县通高速、乡镇通国省道、村村通沥青水泥路、打通出口路”的目标;铁路方面,建成宝兰客运专线,积极推进兰州至天水至汉中城际铁路、天水至成县至武都、天水至成县至阳平关铁路项目,构建天水铁路“十”字形骨架;机场方面,完成天水机场迁建工程。

加强水利能源保障。加快建设城区引洮供水工程,尽早实施曲溪城乡供水、引洮二期工程、张家川东部供水等项目,配合做好白龙江引水项目前期工作,从根本上解决城乡供水紧缺问题。积极争取华电2×350兆瓦和清水2×1000兆瓦火电项目,“十三五”全市所有县区实现天然气管道供应。

打造物流信息中心。加快实施天水商贸城、甘肃东部甘谷物流中心、秦安中国西部小商品城等项目,启动建设天水汽车博览城、秦州藉口物流园、麦积东柯物流园、武山中乾物流园等项目,全力实施天水国际“陆港”项目。推进“互联网+”行动,争取国家和省上布局设立互联网骨干直联点,规划建设“云计算”数据中心,完善地理信息大数据共享交换平台,努力打造数字天水。

四、坚持集约组团发展,统筹推进城市工作,加快建设区域中心城市和现代化大城市

优化城市发展布局。充分发挥规划引领作用,完成县域及中心镇总体规划修编,乡村规划覆盖率达到100%。统筹城市空间、规模、产业三大结构,着力打造“一核、三轴、两区”城镇区域空间布局,加快构建“五区三县”城市框架。按照“东西延伸、相向对接、南北优化、旧城更新”的总体思路,统筹抓好古城保护、旧城更新和新城开发,积极推进西关古城片区保护复兴,加快秦州新城、成纪新城、麦积新城、颖川新城、东柯新城、中梁空港城、三阳川新区、社棠工业城八块新城区建设,按照一体化规划、组团式发展拓展城市空间。

完善城市服务功能。着力提升城市通达能力,加快建设秦州大道、城市轨道交通、藉河罗家沟大桥等项目,启动实施麦积山大道、东柯大道、秦州至三阳川隧道、藉河二十铺大桥等项目。加强城市公共设施建设,加快实施天水会展中心和城区热源厂建设项目,统筹抓好社区市场、公交车停靠点、公共自行车、公共停车场、过街天桥等设施建设,进一步提升市政公共用品和服务供给能力。积极实施城建民生工程,持续做好小巷道整修硬化、人行道铺装改造、公厕改造提升等工作,提高城市综合承载能力。加快推进“海绵城市”和地下综合管廊建设。

提升城市治理水平。转变城市治理方式,优化城市综合管理体制,加快“智慧城市”建设,推进城市管理精细化、现代化和智能化。持续开展“三城联创”活动,下气力解决交通秩序、环境卫生、大气质量、乱搭乱建等诸多“城市病”。优化美化城市生态环境,完成藉河风情线一期延伸和二期启动、“两山两河”百公里生态长廊等项目,积极创建国家园林城市、国家卫生城市。加大棚户区和城中村改造力度,促进房地产业健康发展。

推进新型城镇化建设。坚持以城带乡、城乡互促,重视加强五县县城建设,不断完善道路、供水、供电、供暖、供气、通讯和垃圾处理、污水处理等基础设施,加快县域经济发展,提高县城承载能力。深入开展“一县十镇二十村”新型城镇化试点,加快美丽乡村建设,着力打造一批“小而精、小而美”的特色小镇和风情小镇。深化户籍制度改革,促进农民工常住居民的市民化,鼓励农村转移人口举家进城进镇落户,加快推进以人为核心的新型城镇化建设。“十三五”末,全市常住人口城镇化率达到53%,年均提高2个百分点。

五、坚持全面深化改革,扩大对外交流合作,激发经济社会发展活力和动力

深化重点领域改革。开展农村土地征收、集体经营性建设用地入市、宅基地制度改革试点,全面启动国有林场改革,完成农村土地承包经营权确权登记颁证和甘谷县全国农村供销合作社综合改革试点。深入推进“先照后证”改革,推行市场准入负面清单管理模式。完善国资监管职能,深化国资国企改革。推进投融资体制改革,提升城市建设、交通发展、文化旅游、天源水务、工业资产等市级投融资平台运作水平,推广运用“PPP”等项目建设运营模式,培育发展各类投资基金。推进财税金融改革,加快多层次资本市场发展,扩大直接融资规模,加强金融风险防范。“十三五”末,全市上市企业和在全国性及区域性股权市场挂牌托管企业达到220户以上。

加强区域交流合作。推进关中—天水经济区建设,争取设立关中—天水经济区产业投资基金,开通天水—西安以东“天水号”旅游专列、天水—平凉—中卫—兰州—天水旅游环线列车和天水—敦煌旅游列车,依托西安港、“长安号”国际货运班列,通过“借船出海”等方式融入区域发展大局。提升“公祭伏羲大典”层次和水平,积极申报国家级祭祀。办好环青海湖国际公路自行车赛天水段、中国龙舟公开赛、全国山地自行车冠军赛、国际传统射箭锦标赛等体育赛事,提升天水的影响力和知名度。借助丝绸之路(敦煌)国际文博会、兰洽会等重大节会活动,加强天水特色产品和优势资源的宣传推介,引进一批产业关联度强、示范带动作用大的招商项目落地实施。

提升外贸发展水平。坚持办好中阿共建丝绸之路经济带天水合作交流暨阿拉伯国家驻华使节天水行活动,推动特色农业、装备制造、电工电器、清真食品、民俗民族工艺品等企业与丝路沿线国家和地区开展务实合作,扩大进出口规模。加快天水海关和保税物流中心建设,争取建立综合保税区,推动对外贸易便利化。“十三五”期间外贸进出口总额年均增长5%以上。

六、坚持绿色低碳发展,筑牢生态安全屏障,全面推进生态文明建设

坚持绿色发展理念。加快组建“中国绿色催化专家智库”,精心筹办两年一届的“绿色和可持续发展技术麦积山论坛”,着力打造国家级高端智库和具有国际影响力的绿色可持续发展论坛品牌。依托智库资源,运用论坛成果,加强产学研对接,实施一批绿色低碳产业化项目,孵化一批具有示范引领作用的样板企业。

加强生态环境保护。实施渭河干流天水段及其支流、西汉水等重点区域生态综合治理项目,推进新一轮退耕还林、天然林保护二期、三北五期防护林、国家公益林管护等林业生态工程,积极打造国家生态安全屏障综合试验区“南部基地”,积极争取国家地质灾害治理、土地整理、西秦岭生态环境建设等项目。“十三五”末,全市森林覆盖率达到36%以上,城市建成区绿化覆盖率达到45%以上。大力整治环境污染,建成县级环境空气质量监测点,下决心取缔城区燃煤小锅炉,实现五县县城和重点镇生活污水、垃圾处理设施全覆盖和稳定达标运行,加强河道治理和农村环境整治,全面完成“十三五”污染减排目标任务,推动环境质量持续改善。

积极发展循环经济。加快实施《天水市循环经济发展规划》,积极推进循环经济“五大载体”建设,完善天水装备制造循环经济基地建设,提升经济技术开发区循环化改造水平,推广天水国家农业科技园区循环模式,抓好循环经济示范企业培育和重点项目实施,着力构建“四位一体”循环经济体系。落实节能减排目标考核制度,提高新能源公交车、出租车比例,努力建设天水国家循环经济产业聚集区和全国循环农业示范市。

七、坚持保障改善民生,强化共建共享理念,切实增强人民群众的获得感和幸福感

协调发展社会事业。坚持教育优先发展战略,完成义务教育“全面改薄”任务,着力提升市区教育资源服务水平,实现县域义务教育均衡发展。加快发展学前教育,实施职业教育“22651”计划,普及高中阶段教育,重视加强特殊教育和老年教育,争取天水农校、天水卫校分别创建为职业技术学院,积极创办天水大学和天水理工大学。推动医疗卫生资源向基层倾斜,提升县乡级医院综合服务能力,“十三五”末力争90%以上的病人在县域内就诊。支持发展中医药事业,推进陇东南国家中医药养生保健旅游创新区建设。加快文化体育事业发展,建成市体育中心、文化中心等项目,努力建设文化、旅游、体育大市和强市。

努力扩大城乡就业。高度重视大学生就业工作,落实“援企稳岗”政策,实施新生代农民工职业技能提升计划,开展贫困家庭子女、未升学初高中毕业生、农民工、失业人员和转岗职工、退役军人免费接受职业培训行动,做好公益性岗位托底安置工作。“十三五”全市年均新增城镇就业6万人以上,城镇登记失业率控制在4%以内。

维护社会和谐稳定。实施全民参保计划,落实机关事业单位职工养老保险和城乡居民大病保险制度,做好被征地农民养老保险工作,建成“五险合一”大数据平台,大力发展职业年金、企业年金和商业养老保险。到“十三五”末,城乡居民社会养老保险覆盖率达到95%以上,城镇职工基本医疗保险、城镇居民基本医疗保险、生育保险、工伤保险动态覆盖率达到98%以上。统筹做好社会救助、社会福利和慈善事业。深化双拥共建活动,推动军地深度融合发展。加强和创新社会管理,深入开展“平安天水”、“法治天水”建设,加强社会治安防控体系建设,严厉打击各类违法犯罪活动,有效化解各类社会矛盾。高度重视安全生产,及时防范和处置各类突发问题,确保社会大局和谐稳定。

八、坚持转变政府职能,重视加强自身建设,着力打造人民满意的政府

坚持依法行政。重视地方立法工作,及时向市人大常委会提出立法项目规划和计划建议,重视做好以市政府作为提案人的地方性法规(草案)调研论证起草工作。完善政府法律顾问制度,严格按法定权限和程序履行职责,坚持重大行政决策公众参与、专家论证、风险评估、合法性审查、集体讨论决定等制度,推动决策科学化、民主化、法治化。持续推进全民普法工作,进一步深化政务公开,自觉接受人大工作监督和法律监督、政协民主监督,重视人民群众监督和社会舆论监督,优化改进人大代表建议和政协提案办理方式。

坚持从严治政。严格执行中央“八项规定”、省委“双十条”规定和市委“二十七条”实施意见,持之以恒整治“四风”,带头践行“三严三实”,坚决纠正庸政、懒政、怠政和为官不为现象,强化行政执行力,提升政府公信度。深化行政审批制度改革,加强市、县区政府部门“三张清单一张网”动态管理,实现“网上行权”。推进事业单位分类改革,扩大政府购买服务范围,构建多层次公共服务供给体系。

坚持廉洁从政。认真落实政府系统党风廉政建设“主体责任”和领导干部“一岗双责”,健全重点领域廉政风险防控制度,强化行政监察和审计监督,加强权力运行监督制约。坚持以零容忍态度惩治腐败,严肃查处违纪违法案件,坚决纠正损害群众利益的不正之风。大兴勤俭节约、艰苦奋斗之风,压减“三公”经费支出,树立为民务实清廉的良好形象。

(三)2016年政府工作的主要任务

2016年,是实施“十三五”规划的开局之年,做好今年的工作意义重大。全市经济社会发展的主要预期目标是:生产总值增长8%,规模以上工业增加值增长9%,固定资产投资增长10%,社会消费品零售总额增长10%,公共财政预算收入增长9%,城镇居民人均可支配收入增长9%,农村居民人均可支配收入增长12%,人口自然增长率控制在15.6‰以下,居民消费价格涨幅控制在3%以内,单位生产总值能耗完成省上下达指标。政府工作的主要任务是:

一、凝心聚力抓好重点项目。深入实施“3341”项目工程,年内建成宝兰客运专线、麦甘二级公路、武山贺家店至岷县战备公路、麦积客运站、城区应急供水等重点项目;加快推进国道310秦州至武山段升级改造、高铁南站综合交通枢纽及站前广场、天水汽车西站、甘谷客运中心、清水汽车站、城区引洮供水、张家川富川水库、秦安县小湾河水库、秦州关峡水库等项目;启动实施平凉至天水高速公路、陕西陇县至榜罗公路秦安至甘谷段、天定高速藉口出口、天巉公路“三站合一”中滩收费站等重点项目以及甘泉至麦积山一级公路、洛门至水帘洞二级公路、温泉至虹门子3条旅游公路;积极推进天水机场迁建、静宁至天水高速公路、天水南绕城高速、陕西陇县至榜罗公路清水至秦安段、天水至武都铁路、兰州至天水至汉中城际铁路、曲溪城乡供水等项目前期工作。全面实施财政易地搬迁项目,加快秦州区藉口镇、平南镇和麦积区颖川佳苑、高铁佳苑等13处搬迁安置点建设,打造一批特色小镇和风情小镇。加大招商引资力度,积极推进万达广场等项目落地实施,力争招商引资到位资金增长12%以上。

二、全面提速推进城市建设。完成第四版天水市城市总体规划纲要编制。积极争取把天水纳入正在规划建设的国家城市群名单。加快八块新城区开发建设,成纪新城年内初具规模,颖川新城、东柯新城文化旅游项目实现重点突破,秦州新城基础设施项目得到有效实施,其他新城区建设都有新的进展。加强城区基础设施建设,完成成纪大道绿化亮化景观工程、商贸城路网、藉河双桥大桥、藉河罗峪河口大桥等项目,加快建设秦州大道、藉河罗家沟大桥、有轨电车一号线、成纪新城地下净水厂、秦州热源厂、天水固体垃圾处理循环产业园等项目,启动建设麦积山大道、东柯大道、秦州至三阳川隧道、成纪新城路网、岷山厂片区路网、体育中心路网、藉河南路西延段二期、王家磨景观廊桥、成纪新城热源厂、渭河峡口段生态环境综合治理、城区供热供水供气管网改造等项目,积极推进“海绵城市”建设,在新区开发中探索建设“地下综合管廊”。加快实施棚户区改造项目,加大城中村改造力度,促进城市重点片区开发建设。加强历史文化名城保护和城区绿化美化,加快建设藉河风情线一期延伸项目,启动实施藉河风情线二期、人民公园改造、天水名城博物馆等项目。重视加强城市管理,推进“智慧城市”试点工作,加快“三城联创”步伐,依法从严查处乱搭乱建、乱停乱放、乱堆乱倒、占道经营、违法建设等行为,有效解决城区交通堵、停车难、秩序乱、环境差等“城市病”。

三、提质扩量促进工业发展。打好战略性新兴产业培育攻坚战,加大对重点行业和21户省市级骨干企业扶持力度,全力打造六大百亿元产业集群。积极推进开发区扩区增容,加快实施长城电工电器产业园、华天科技产业园二期、电工产品综合检测实验研究基地、长城果汁10万吨果蔬汁罐装示范基地、天河酒业生态酿酒城技改扩建、胜华电缆、武山干法水泥生产线等重点项目,盯紧落实天水烟用包装印刷材料、大型客机改货机、长控公司和铁路电缆出城入园等有一定基础的项目。推动华洋电子、华圆科技、星火机床、海林中科、锻压机床等企业上市融资或在“新三板”挂牌。抓住国家“去产能”的政策机遇,加强企业资产重组、产权转让等改革。建设“绿色和可持续发展技术麦积山论坛”永久性会址,全力办好首届“麦积山论坛”,积极承接绿色可持续产业化项目。加快创建全国质量强市示范城市,重视加强标准化工作,设立标准创新贡献奖,年内创建省级以上企业技术中心1家、省级技术创新示范企业2家,甘肃名牌产品8个,中国驰名商标2件。

四、聚焦脱贫做好“三农”工作。深入推进精准脱贫,规范建档立卡工作,完善精准扶贫大数据平台,加强扶贫互助协会规范化建设,全力抓好产业培育、安全饮水、自然村通动力电、易地搬迁、危房改造等重点工作,力争财政专项扶贫资金突破5亿元,整合扶贫资金120亿元,开展扶贫培训3万人次,实施整村推进项目140个以上,年内减贫16万人,力争两个县区实现整体脱贫。落实科技增粮措施,确保正常年景粮食总产达到120万吨以上。新建特色林果基地25万亩,新增优质蔬菜5.6万亩,新建标准化规模养殖场100个,有组织输转劳动力70万人,果品、蔬菜、畜牧三大产业产值分别达到90亿元、68亿元和70亿元,劳务收入达到120亿元。完成造林20万亩,修建堤防60公里,兴修梯田15万亩,治理水土流失面积300平方公里。

五、综合施策提升三产水平。实施电子商务进农村综合示范项目,完成天水众创空间暨电子商务中心建设,积极争创国家级电子商务示范基地。规划建设天水国际“陆港”,建成公用型保税仓库和出口监管仓库,力争外贸进出口总额达到25亿元以上。加快商贸物流设施建设,推进天水商贸城等项目,完成肉类蔬菜流通追溯体系项目一期工程。编制完成《麦积山风景名胜区总体规划》,改革麦积山大景区管理体制,力争全年旅游接待人数、综合收入分别增长27.5%、28.2%。精心筹办公祭伏羲大典暨第27届伏羲文化旅游节,办好第37届“花牛苹果·向阳杯”全国少儿乒乓球比赛和影像丝绸之路天水摄影双年展。加强与关中—天水经济区、丝绸之路经济带沿线城市的交流联系,深化经贸、文化、旅游等领域合作。加大非公经济扶持力度,非公经济增加值增长11%以上。

六、统筹协调发展社会事业。重视发展教育事业,实施“全面改薄”项目1080个,建设校舍40.5万平方米,完成市一中麦积校区建设,加快实施市三中迁建项目,新建扩建市逸夫中学、市五中、市七中、麦积区润天学校、秦州区玉泉中学5所初中教学楼和秦州区山水嘉园小学、麦积区区府路小学2所小学,改建新建城区10所幼儿园。实施行政村幼儿园建设项目343个,实现1500人以上有实际需求的行政村学前教育全覆盖。推进创业创新平台建设,争取国列、省列科技项目40项以上。重视文化体育基础设施建设,加快建设市体育中心,建成乡村舞台529个、历史再现博物馆50个,新建改建旅游厕所90个,全力承办好全省第三届中学生运动会及各项体育赛事活动。积极推进县级公立医院综合改革,完成市中心血站、市妇幼保健院搬迁项目,不断改善医疗卫生条件。坚持计划生育基本国策,实施“全面二孩”政策。

七、多措并举促进社会和谐。积极推进就业创业,年内新增城镇就业5.6万人,城镇登记失业率控制在4%以内。落实不动产登记政策,全面完成公车改革任务。严格落实“党政同责、一岗双责、齐抓共管”的安全生产工作机制,加强重点领域和重点行业隐患排查整治,严防各类安全生产事故。认真落实信访工作责任制,及时办理答复群众通过“市委书记市长留言板”、“市长信箱”等渠道反映的问题。加大农产品和食品药品监管力度,确保群众饮食用药安全。深化“平安天水”建设,加强防暴处突和反恐维稳工作,重视做好严重精神障碍患者救治管理,强化重大金融风险防控,严厉打击网络诈骗、电信诈骗和非法集资等犯罪活动。依法管理民族宗教事务,高度重视民族地区和民族经济发展,努力营造和谐稳定的社会环境。

八、倾心尽力办好民生实事。集中力量抓好省、市确定的民生实事,确保按期全部兑现落实。(1)扶持1000名以上普通高校毕业生到企业、基层一线就业。(2)城市低保标准提高10%,农村一、二类低保实现与扶贫脱贫线“两线合一”,农村五保供养补助标准提高15%,城乡孤儿基本生活补助实现统一标准。(3)在省外建立果蔬及土特产品直销店10个,加大果品、蔬菜宣传推介力度,让天水“花牛苹果”等农产品品牌“更牛”。(4)年内实现村村通沥青(水泥路)、村村通安全饮水、村村通动力电、村村通有线电视以及4G互联网络全覆盖。(5)实施城建民生工程,在市区建成1000辆公共自行车租赁系统,改造提升200个公交站点,新增5000个停车位,全市完成10万平方米的人行道透水铺装改造,完成100条小巷道综合整治,改造提升公厕50座,建成300个绿地花坛。(6)改造提升人民公园,完成马跑泉公园改造。(7)实施棚户区改造1.7万套。(8)实施城区教育资源提升“2521计划”,续建新建改建高级中学2所、初级中学5所、小学2所、幼儿园10所。(9)对贫困县区乡村中小学、幼儿园教师发放生活补助,对学前教育幼儿免保教费,对建档立卡贫困家庭普通高中学生免学杂费和书本费。(10)新建乡镇及社区体育健身中心10个、行政村农民体育健身工程60个。(11)在房产项目中代建或在城区建设5个小吃市场,逐步解决城区小吃摊点随意乱摆和占路为市的问题。(12)改造和取缔城区小锅炉,开展集中供热和清洁能源供热,加大水泥厂等企业环境监管力度,加强城乡结合部环境污染治理,让“天水蓝”更蓝。

各位代表,“十三五”发展的宏伟蓝图已经绘就,我们承担的历史使命十分光荣,面临的工作任务异常艰巨。我们要在省委、省政府和市委的坚强领导下,团结带领全市人民,开拓创新,真抓实干,勇于担当,主动作为,扎扎实实地做好今年的工作,努力实现“十三五”经济社会发展的良好开局,为与全国全省一道全面建成小康社会而努力奋斗!
